# Family-group names for termites (Isoptera), redux

**DOI:** 10.3897/zookeys.148.1682

**Published:** 2011-11-21

**Authors:** Michael S. Engel

**Affiliations:** 1 Division of Entomology, Natural History Museum, and Department of Ecology & Evolutionary Biology, 1501 Crestline Drive – Suite 140, University of Kansas, Lawrence, Kansas 66049-2811, USA

**Keywords:** Isoptera, termites, nomenclature, classification, family-group names, type genera

## Abstract

Forty-eight family-group names are identified for insects among the Isoptera (termites), representing a nearly 19% increase since the last compilation less than 10 years ago. Accordingly, these names are newly catalogued, including various updates from the original summary. The name Reticulitermitidae is recognized as a *nomen nudum* while Caatingatermitinae is newly considered a *nomen invalidum*, and neither is available in zoological nomenclature. A catalogue of the suprafamilial names for Isoptera is appended. The name Xylophagodea is formally proposed for the Isoptera + Cryptocercidae clade.

## Introduction

During the last 25 years numerous efforts have been undertaken to document family-group names for insects and to provide their correct authorship, date, type genus, combining stem, and availability or validity (e.g., Michener 1986; Wahl and Mason 1995; Menke 1997; Sabrosky 1999; Speidel and Naumann 2004; Engel 2005; Engel and Haas 2007; Menke et al. 2008; Miller 2009; Bouchard et al. 2011). Seven years ago such an exercise was completed for the termites (Engel and Krishna 2004a) and it is remarkable that the number of names has grown significantly for this small insect lineage, of approximately 3500 species, such that a full 19% of the names known today were not included in that original account. Accordingly, I provide an updated list, in order of priority, for all family-group names proposed for termites through to the present day. The list is an updated and corrected version of that provided by Engel and Krishna (2004a), incorporating the various new names and edits of subsequent works (e.g., Engel and Krishna 2004b, 2007; Cancello and DeSouza 2005; Engel et al. 2009). As in the earlier summary, all names are presented in their original forms, regardless of present day rank or suffix, with type genus and correct combining stem provided for each. Daggers (†) indicate names proposed for fossil taxa. The format generally follows that of Engel and Krishna (2004a).

In addition I have included here for the first time a summary of all names applied for suprafamilial groups of termites (i.e., names above the family-group ranks and not regulated by the ICZN).

## Catalog of Family-group names

1. **Termitina** Latreille, 1802: 293. Type genus: *Termes* Linnaeus, 1758. Combining stem: Termit–. Note: Latreille (1805, 1810) subsequently changed the name of his “famille” to Termitinae but the name was made first available in 1802 (ICZN 1999: Art. 11.7).

2. **Calotermitinae** Froggatt, 1897: 516 [*recte* Kalotermitinae; in accordance with ICZN (1999) Art. 29.1 the name is automatically emended to Kalotermitinae Froggatt, 1897]. Type genus: *Kalotermes* Hagen, 1853 [*Calotermes* Hagen, 1858 is an unjustified emendation: *vide* Engel and Krishna 2001a; ICZN 2002]. Combining stem: Kalotermit–.

3. **Glyptotermitinae** Froggatt, 1897: 518. Type genus: *Glyptotermes* Froggatt, 1897. Combining stem: Glyptotermit–.

4. **Rhinotermitinae** Froggatt, 1897: 518. Type genus: *Rhinotermes* Hagen, 1858. Combining stem: Rhinotermit–.

5. **Heterotermitinae** Froggatt, 1897: 550. Type genus: *Heterotermes* Froggatt, 1897. Combining stem: Heterotermit–.

6. **Mastotermitinae** Desneux, 1904a: 284. Type genus: *Mastotermes* Froggatt, 1897. Combining stem: Mastotermit–.

7. **Hodotermitini** Desneux, 1904c: 284. Type genus: *Hodotermes* Hagen, 1853. Combining stem: Hodotermit–.

8. **Stolotermitinae** Holmgren, 1910a: 285. Type genus: *Stolotermes* Hagen, 1858. Combining stem: Stolotermit–. Note: Stolotermitinae has often been included in an expanded Termopsidae for which the latter name has been used for the combined grouping despite the priority of the name based on *Stolotermes*. A petition was submitted (Engel et al. 2003; Engel and Krishna 2004c) and approved (ICZN 2005) reversing priority in favor of Termopsidae whenever *Termopsis* and *Stolotermes* are placed within the same family group. However, in the present classification (Engel et al. 2009 and table 1 herein) stolotermitines are segregated into their own family.

9. **Leucotermitinae** Holmgren, 1910a: 285. Type genus: *Leucotermes* Silvestri, 1901. Combining stem: Leucotermit–.

10. **Coptotermitinae** Holmgren, 1910a: 285. Type genus: *Coptotermes* Wasmann, 1896. Combining stem: Coptotermit–. Note: Proposed again as new in Holmgren (1910b).

11. **Serritermitinae** Holmgren, 1910a: 285. Type genus: *Serritermes* Wasmann, 1897. Combining stem: Serritermit–.

12. **Termitogetoninae** Holmgren, 1910a: 286. Type genus: *Termitogeton* Desneux, 1904b. Combining stem: Termitogeton–.

13. **Microcerotermitinae** Holmgren, 1910b: 145. Type genus: *Microcerotermes* Silvestri, 1901. Combining stem: Microcerotermit–.

14. **Eutermitinae** Holmgren, 1910b: 146. Type genus: *Eutermes* Heer, 1849. Combining stem: Eutermit–. Note: Given the uncertainty in the application of the name *Eutermes*, and thereby the family-group name Eutermitinae, a petition to suppress proactively the name was submitted to the ICZN for consideration (Engel and Krishna 2005a) which, after discussion (Roisin 2005; Engel and Krishna 2005b), was not approved (ICZN 2007). The name is presently considered *incertae sedis*.

15. **Termopsinae** Holmgren, 1911: 35. Type genus: *Termopsis* Heer, 1849. Combining stem: Termops–. Note: Refer to comments provided for Stolotermitinae (*vide supra*).

16. **Psammotermitinae** Holmgren, 1911: 64. Type genus: *Psammotermes* Desneux, 1902. Combining stem: Psammotermit–.

17. **Pseudomicrotermitinae** Holmgren, 1912: 5. Type genus: *Pseudomicrotermes* Holmgren, 1912. Combining stem: Pseudomicrotermit–.

18. **Foraminitermitinae** Holmgren, 1912: 5. Type genus: *Foraminitermes* Holmgren, 1912. Combining stem: Foraminitermit–.

19. **Stylotermitinae** Holmgren and Holmgren, 1917: 141. Type genus: *Stylotermes* Holmgren and Holmgren, 1917. Combining stem: Stylotermit–.

20. †**Pliotermitinae** Pongrácz 1917: 28. Type genus: †*Pliotermes* Pongrácz, 1917. Combining stem: Pliotermit–.

21. **Arrhinotermitinae** Sjöstedt, 1926: 8. Type genus: *Arrhinotermes* Wasmann, 1902. Combining stem: Arrhinotermit–.

22. **Acanthotermitinae** Sjöstedt, 1926: 8. Type genus: *Acanthotermes* Sjöstedt, 1900. Combining stem: Acanthotermit–. Note: This name has priority over Macrotermitinae; however, a petition was submitted to conserve the usage of Macrotermitinae (Engel and Krishna 2001b) and was approved by the ICZN (2003). Macrotermitinae is to be used whenever *Macrotermes* and *Acanthotermes* are placed into the same family-group taxon.

23. †**Miotermitinae** Pongrácz, 1926: 29 [chart]. Type genus: †*Miotermes* Rosen, 1913. Combining stem: Miotermit–.

24. **Macrotermitinae** Kemner, 1934: 69. Type genus: *Macrotermes* Holmgren, 1909. Combining stem: Macrotermit–. Note: Refer to comments provided for Acanthotermitinae (*vide supra*).

25. **Amitermitinae** Kemner, 1934: 110. Type genus: *Amitermes* Silvestri, 1901. Combining stem: Amitermit–.

26. **Miro-capritermitinae** Kemner, 1934: 166. Type genus: *Mirocapritermes* Holmgren, 1914. Combining stem: Mirocapritermit–. Note: Although Kemner (1934) hyphenated the name in its original spelling, the ICZN (1999) does not allow hyphenation and the family-group name must be considered a single word.

27. **Nasutitermitinae** Hare, 1937. Type genus: *Nasutitermes* Dudley, 1890. Combining stem: Nasutitermit–.

28. †**Electrotermitinae** Emerson, 1942: 10. Type genus: †*Electrotermes* Rosen, 1913. Combining stem: Electrotermit–.

29. **Porotermitinae** Emerson, 1942: 10. Type genus: *Porotermes* Hagen, 1858. Combining stem: Porotermit–.

30. **Apicotermitinae** Grassé and Noirot, 1954 [1955]: 360. Type genus: *Apicotermes* Holmgren, 1912. Combining stem: Apicotermit–.

31. **Odontotermitini** Weidner, 1956: 82. Type genus: *Odontotermes* Holmgren, 1910a. Combining stem: Odontotermit–.

32. **Cubitermitini** Weidner, 1956: 99. Type genus: *Cubitermes* Wasmann, 1906. Combining stem: Cubitermit–.

33. **Mirotermitini** Weidner, 1956: 99. Type genus: *Mirotermes* Wasmann, 1897. Combining stem: Mirotermit–.

34. **Capritermitini** Weidner, 1956: 100. Type genus: *Capritermes* Wasmann, 1897. Combining stem: Capritermit–.

35. **Indotermitidae** Roonwal and Sen-Sarma *In* Roonwal, 1958: 81. Type genus: *Indotermes* Roonwal and Sen-Sarma *In* Roonwal, 1958. Stem: Indotermit–. Note: Proposed as new again in Roonwal and Sen-Sarma (1960).

36. †**Cretatermitinae** Emerson, 1967 [1968]: 278. Type genus: †*Cretatermes* Emerson, 1967 [1968]. Combining stem: Cretatermit–.

37. **Prorhinotermitinae** Quennedey and Deligne, 1975: 265. Type genus: *Prorhinotermes* Silvestri, 1909. Combining stem: Prorhinotermit–.

38. †**Lutetiatermitinae** Schlüter, 1989: 61. Type genus: †*Lutetiatermes* Schlüter, 1989. Combining stem: Lutetiatermit–.

39. †**Carinatermitinae** Krishna and Grimaldi, 2000: 134. Type genus: †*Carinatermes* Krishna and Grimaldi, 2000. Combining stem: Carinatermit–.

40. †**Archeorhinotermitinae** Krishna and Grimaldi, 2003: 2. Type genus: †*Archeorhinotermes* Krishna and Grimaldi, 2003. Combining stem: Archeorhinotermit–.

41. **Reticulitermatidae** Szalanski, Austin, and Owen 2003: 1514, *nomen imperfectum* [*recte* Reticulitermitidae] *et* *nomen nudum*. Note: This name has appeared in several publications (e.g., Sobti et al. 2009) but has not been formally established. It would represent simply a junior synonym of Heterotermitinae.

42. **Syntermitinae** Engel and Krishna, 2004a: 6. Type genus: *Syntermes* Holmgren, 1909. Combining stem: Syntermit–.

43. **Sphaerotermitinae** Engel and Krishna, 2004a: 6. Type genus: *Sphaerotermes* Holmgren, 1912. Combining stem: Sphaerotermit–.

44. **Cornitermitinae** Ensaf et al., 2004: 284, *nomen nudum*. Type genus: *Cornitermes* Wasmann, 1897. Combining stem: Cornitermit–.

45. **Glossotermitinae** Cancello and DeSouza, 2005: 35. Type genus: *Glossotermes* Emerson, 1950. Combining stem: Glossotermit–.

46. †**Caatingatermitinae** Martins-Neto, Ribeiro-Júnior, and Prezoto, 2006: 127, *nomen invalidum*. Note: I consider this name to be unavailable as the type genus was not explicitly indicated. ICZN (1999) Art. 16.2 requires that after 1999 all new family-group names must have the type genus precisely identified, not implied through the formation of the name. Martins-Neto et al. (2006) nowhere mention the type genus for their new subfamily and include two genera and two species within their grouping. Accordingly, this name fails to meet all of the criteria for availability.

47. †**Cratomastotermitidae** Engel, Grimaldi, and Krishna, 2009: 9. Type genus: †*Cratomastotermes* Bechly, 2007. Combining stem: Cratomastotermit–.

48. **Archotermopsidae** Engel, Grimaldi, and Krishna, 2009: 11. Type genus: *Archotermopsis* Desneux, 1904d. Combining stem: Archotermops–.

**Table 1.** Hierarchical and synonymic outline of termite classification (modified after Engel et al. 2009). *Nomina nuda* and *nomina invalida* omitted.

Infraorder **Isoptera** Brullé, 1832					
			Family †Cratomastotermitidae Engel et al., 2009		
	Parvorder Euisoptera Engel et al., 2009				
					*†Cretatermitinae* Emerson, 1967 [1968]
					*†Lutetiatermitinae* Schlüter, 1989
					*†Carinatermitinae* Krishna & Grimaldi, 2000
			Family Mastotermitidae Desneux, 1904a		
					= *†Pliotermitinae* Pongrácz, 1917
					= *†Miotermitinae* Pongrácz, 1926
			Family †Termopsidae Holmgren, 1911		
			Family Hodotermitidae Desneux, 1904c		
			Family Archotermopsidae Engel et al., 2009		
			Family Stolotermitidae Holmgren, 1910a		
				Subfamily Porotermitinae Emerson, 1942	
				Subfamily Stolotermitinae Holmgren, 1910a	
			Family Kalotermitidae Froggatt, 1897		
					= *Glyptotermitinae* Froggatt, 1897
					= *†Electrotermitinae* Emerson, 1942
		Nanorder Neoisoptera Engel et al., 2009			
			Family †Archeorhinotermitidae Krishna & Grimaldi, 2003		
			Family Stylotermitidae Holmgren & Holmgren, 1917		
			Family Rhinotermitidae Froggatt, 1897		
				Subfamily Coptotermitinae Holmgren, 1910a	
					= *Arrhinotermitinae* Sjöstedt, 1926
				Subfamily Heterotermitinae Froggatt, 1897	
					= *Leucotermitinae* Holmgren, 1910a
				Subfamily Prorhinotermitinae Quennedey & Deligne, 1975	
				Subfamily Psammotermitinae Holmgren, 1911	
				Subfamily Termitogetoninae Holmgren, 1910a	
				Subfamily Rhinotermitinae Froggatt, 1897	
			Family Serritermitidae Holmgren, 1910a		
					= *Glossotermitinae* Cancello and DeSouza, 2005
			Family Termitidae Latreille, 1802		
				Subfamily Apicotermitinae Grassé & Noirot, 1954 [1955]	
					= *Indotermitidae* Roonwal & Sen Sarma in Roonwal, 1958
				Subfamily Foraminitermitinae Holmgren, 1912	
					= *Pseudomicrotermitinae* Holmgren, 1912
				Subfamily Sphaerotermitinae Engel & Krishna, 2004a	
				Subfamily Macrotermitinae Kemner, 1934, nomen protectum [ICZN 2003]	
					= *Acanthotermitinae* Sjöstedt, 1926, nomen rejiciendum [ICZN 2003]
					= *Odontotermitini* Weidner, 1956
				Subfamily Syntermitinae Engel & Krishna, 2004a	
					= *Cornitermitinae* Ensaf et al., 2004, nomen nudum
				Subfamily Nasutitermitinae Hare, 1937	
				Subfamily Cubitermitinae Weidner, 1956	
				Subfamily Termitinae Latreille, 1802	
					= *Microcerotermitinae* Holmgren, 1910b
					= *Amitermitinae* Kemner, 1934
					= *Mirocapritermitinae* Kemner, 1934
					= *Mirotermitini* Weidner, 1956
					= *Capritermitini* Weidner, 1956
	*Incertae Sedis*				
					Eutermitinae Holmgren, 1910b

### Catalog of Names above the Family Group

Here I provide a brief checklist of those names applied to termites above the family-group category. While I have included those supraordinal names which combined termites within an expanded taxon alongside one other group of insects (e.g., Aetioptera Enderlein, 1909), I have not listed those older names which united Isoptera with what are today recognized as numerous other orders (e.g., Platyptera Packard, 1883, for Isoptera, Embiodea, Plecoptera, and Psocoptera). In older literature it is often challenging to determine at what rank a particular name was intended or to what categorical level such a name might be equivalent to today. When it has appeared that a name was intended as a family or category loosely equivalent to today’s family group ranks, I have not included it here. For example, the ‘Termitida’ of Haeckel (1866) was as a family of his order Tocoptera, suborder Pseudoneuroptera, section Corrodentia, despite it having a termination reminiscent of that used in other literature as an ordinal, or other suprafamilial, suffix. Accordingly, I do not consider the Termitida of Haeckel (1866) to be the same as the Termitina or Termitida of Krausse (1906a, 1906b) and Krausse and Wolff (1919), since the former was clearly a family-group name [and thereby a *nomen translatum*, whether intentional or not, of Latreille’s (1802) Termitina], while the latter two were explicitly employed as ordinal names. Lastly, despite the considerable biological significance of, and increasing reference in the literature to, the combined Isoptera + Cryptocercidae clade, a name has not formally been proposed for this lineage. Herein I offer the name Xylophagodea for this important biological grouping.

1. **Isoptères** Brullé, 1832: 66 [Latinized by Brauer 1885].

2. **Orthoptera socialia** Gerstaecker, 1863: 40. Originally deemed a ‘guild’ or ‘fraternity’ (“Zunft”, conceptually equivalent in his system to a suborder); equivalent to Isoptera.

3. **Socialia** Börner, 1904: 526. Originally deemed a suborder; equivalent to Isoptera of today [Note: Börner’s ‘Isoptera’ included both Embiodea (as suborder Oligoneura Börner, 1904) and Isoptera (as Socialia *auctorum*)].

4. **Termiten** Krausse, 1906a: 116. Originally deemed an order; equivalent to Isoptera.

5. **Aetioptera** Enderlein, 1909: 171. Originally deemed a superorder; equivalent to Isoptera + Embiodea [as Embiidina].

6. **Cryptoclidoptera** Enderlein, 1909: 171. Originally deemed a suborder; equivalent to all Isoptera excluding Mastotermitidae.

7. **Hemiclidoptera** Enderlein, 1909: 172. Originally deemed a suborder; equivalent to Mastotermitidae.

8. **Termitida** Krausse and Wolff, 1919: 159 [*vide etiam* Rohdendorf 1977]. Equivalent to Isoptera, a form simultaneously used and preferred by the authors.

9. **Termitodea** Kevan, 1977: 12. Originally deemed a suborder; equivalent to Isoptera + Puknoblattinidea Kevan, 1977 [Note: The latter was simultaneously deemed an infraorder for “Puknoblattinidae Sellards, 1908”, although Sellards (1908) never established a family-group for his genus *Puknoblattina*. In considering this genus as the sister group to Isoptera, Kevan (1977) was apparently following the notion of Tillyard (1936).].

10. **Termitidea** Kevan, 1977: 12. Originally deemed an infraorder; equivalent to Isoptera.

11. **Isopterodea** Boudreaux, 1979: 217. Originally deemed a superorder; equivalent to Isoptera as it was the only included order.

12. **Afontanella** Myles, 1998: 334. Originally deemed a suborder; equivalent to Mastotermitidae, Termopsidae s.l., Hodotermitidae, and Kalotermitidae (obviously paraphyletic).

13. **Fontanella** Myles, 1998: 334. Originally deemed a suborder; equivalent to Rhinotermitidae, Serritermitidae, and Termitidae.

14. **Octatubula** Myles, 1998: 334. Originally deemed an infraorder; equivalent to Rhinotermitidae and Serritermitidae.

15. **Quadritubula** Myles, 1998: 334. Originally deemed an infraorder; equivalent to Termitidae.

16. **Euisoptera** Engel, Grimaldi, and Krishna, 2009: 3. Originally rankless; equivalent to all Isoptera exclusive of Cratomastotermitidae and Mastotermitidae.

17. **Neoisoptera** Engel, Grimaldi, and Krishna, 2009: 9. Originally rankless; equivalent to clade comprising Stylotermitidae, Rhinotermitidae, Serritermitidae, and Termitidae.

18. **Xylophagodea**, herein. Originally rankless; equivalent to clade comprising Isoptera and Cryptocercidae.
